# Overexpression of *lpxT* Gene in *Escherichia coli* Inhibits Cell Division and Causes Envelope Defects without Changing the Overall Phosphorylation Level of Lipid A

**DOI:** 10.3390/microorganisms8060826

**Published:** 2020-05-30

**Authors:** Federica A. Falchi, Flaviana Di Lorenzo, Roberto Pizzoccheri, Gianluca Casino, Moira Paroni, Francesca Forti, Antonio Molinaro, Federica Briani

**Affiliations:** 1 Dipartimento di Bioscienze, Università degli Studi di Milano, 20133 Milano, Italy; federica.falchi@unimi.it (F.A.F.); roberto.pizzoccheri@studenti.unimi.it (R.P.); gianluca.casino@studenti.unimi.it (G.C.); moira.paroni@unimi.it (M.P.); francesca.forti@unimi.it (F.F.); 2 Dipartimento di Scienze Chimiche, Università degli Studi di Napoli Federico II, 80126 Napoli, Italy; flaviana.dilorenzo@unina.it (F.D.L.); molinaro@unina.it (A.M.)

**Keywords:** membrane proteins, outer membrane, peptidoglycan, lipopolysaccharide (LPS), lipid A phosphorylation, lipid A modification, LPS transport, LpxT, L,D-transpeptidases

## Abstract

LpxT is an inner membrane protein that transfers a phosphate group from the essential lipid undecaprenyl pyrophosphate (C-55PP) to the lipid A moiety of lipopolysaccharide, generating a lipid A *tris*-phosphorylated species. The protein is encoded by the non-essential *lpxT* gene, which is conserved in distantly related Gram-negative bacteria. In this work, we investigated the phenotypic effect of *lpxT* ectopic expression from a plasmid in *Escherichia coli*. We found that *lpxT* induction inhibited cell division and led to the formation of elongated cells, mostly with absent or altered septa. Moreover, the cells became sensitive to detergents and to hypo-osmotic shock, indicating that they had cell envelope defects. These effects were not due to lipid A hyperphosphorylation or C-55PP sequestering, but most likely to defective lipopolysaccharide transport. Indeed, *lpxT* overexpression in mutants lacking the L,D-transpeptidase LdtD and LdtE, which protect cells with outer membrane defects from osmotic lysis, caused cell envelope defects. Moreover, we found that pyrophosphorylated lipid A was also produced in a *lpxT* deletion mutant, indicating that LpxT is not the only protein able to perform such lipid A modification in *E. coli*.

## 1. Introduction

The envelope of Gram-negative bacteria is a highly organized structure composed of an inner and an outer membrane (IM and OM, respectively) separated by an aqueous space, the periplasm, which contains a thin layer of peptidoglycan (PG) [1]. The synthesis of all layers composing the envelope must be tightly coordinated to allow the cells to maintain their integrity and morphology during growth and division [1,2,3].

The PG, or murein, is a mesh-like macromolecule with a major role in defining cell shape and resistance to osmotic shock. It is organized in parallel glycan strands deriving from the polymerization of an *N*-acetylglucosamine-*N*-acetylmuramic acid (NAG-NAM) disaccharide unit [4]. The NAG-NAM filaments are kept together by amide bonds occurring between short peptide stems covalently linked to the carboxyl carbon of NAM residues located in adjacent filaments [4]. During PG biosynthesis, the NAG-NAM unit is linked to the undecaprenyl phosphate (C55-P), which is an essential carrier with a lipid tail embedded in the IM. As it is, the NAG-NAM unit is flipped from the inner to the outer face of the IM. Then, proteins with transglycosylase activity promote the polymerization of NAG-NAM into the glycan strands, releasing the C55-P carrier in its pyrophosphorylated form (C55-PP). The amide bonds bridging the glycans strands are formed by penicillin-binding proteins (PBP) between the amino acids at position 4 and 3 of the oligopeptides linked to NAM residues [4]. Besides the 4,3 crosslinks, a small proportion of 3,3 crosslinks has also been observed in the PG [5]. Formation of 3,3 crosslinks is catalyzed by penicillin-insensitive L,D-transpeptidases, whose activity has been associated to resistance to β-lactam antibiotics in both Gram-positive and Gram-negative bacteria [6,7]. 

**Figure 1:**
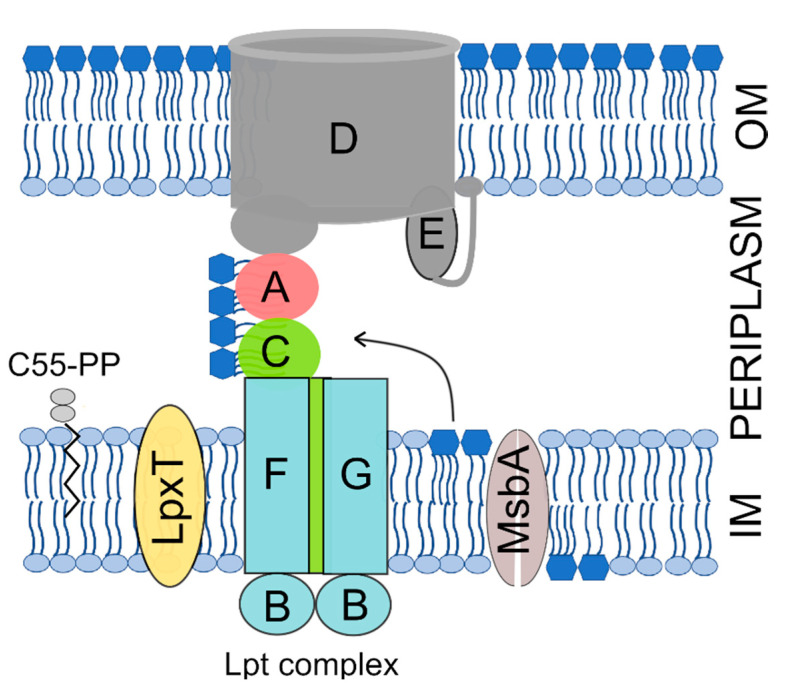
The Gram-negative cell envelope and factors involved in lipopolysaccharide (LPS) transport and phosphorylation mentioned in this work. The inner membrane (IM) and outer membrane (OM) are separated by the periplasmic space, which contains the peptidoglycan (not represented). The Lpt complex subunits are indicated by capital letters. The LPS core oligosaccharide has been omitted from the representation for simplicity. C55-PP, undecaprenyl pyrophosphate; A, LptA; B, LptB; C, LptC; D, LptD; E, LptE; F, LptF; G, LptG.

The OM is an asymmetric bilayer with an inner and an outer leaflet respectively composed of phospholipids and of a peculiar glycolipid, the lipopolysaccharide (LPS) [8]. The LPS consists of an acylated and phosphorylated glucosamine disaccharide named lipid A to which the oligosaccharide core and a long and variable polysaccharide, the O-antigen, are linked. The lipid A-core oligosaccharide is synthesized in the IM inner leaflet and flipped to the IM periplasmic leaflet by the MsbA ATP-binding cassette (ABC) transporter. The O-antigen is then ligated to the core and the LPS is translocated to the OM outer leaflet by the multiprotein Lpt machinery, which spans all envelope layers [9] (Figure 1). 

Although the lipid A is the most conserved part of the LPS, several chemical modifications in its fine structure can be observed [10]. In particular, the phosphate groups of the glucosamine disaccharide can be decorated with positively charged residues, such as 4-amino-4-deoxy-L-arabinose (L-Ara4N) and phosphoethanolamine (PEtN), or with an additional phosphate at position 1, which is the reducing glucosamine unit, forming a *tris*-phosphorylated lipid A species [11]. It has been reported that in *Escherichia coli*, lipid A pyrophosphorylation depends on the activity of the LpxT protein that exploits C55-PP as phosphate donor [12]. Since only the C55-P species can be loaded with the NAG-NAM PG unit (and other non-essential molecules), C55-PP dephosphorylation is required both to recycle this lipid after the delivery of its cargo and also during the C55-P de novo synthesis, as the undecaprenyl pyrophosphate synthase UppS generates the pyrophosphorylated C55-PP form [13,14]. Besides LpxT, three other phosphatases (namely, UppP, PgpB, and YbjG), all located in the IM, participate in the C55-PP conversion into C55-P. These enzymes are partially redundant, as the production of one out of them is sufficient to sustain *E. coli* growth. On the contrary, LpxT alone cannot replace the absence of all other enzymes [12,15,16]. 

LpxT is an integral membrane protein with the catalytic site facing the periplasm that belongs to the conserved family of the PAP2 acid phosphatases [13,16]. In *E. coli*, LpxT is encoded by the *lpxT* gene and it is subject to a complex regulation. Indeed, *lpxT* transcription initiation is downregulated at 37–42 °C vs. 30 °C, in stationary phase and at high Mg^2+^ concentration [15,17,18]. The *lpxT* mRNA stability is also modulated, as the mRNA is quickly destabilized after a temperature upshift from 30 to 37 °C [17]. The physiological meaning of *lpxT* regulation in response to temperature changes remains to be established. LpxT activity is negatively regulated by the PmrR protein in response to different stimuli such as high iron or acid pH [19,20,21]. 

In previous studies, we noticed that the expression of *E. coli lpxT* from a plasmid inhibited bacterial growth, as also previously reported [15]. However, expression from a plasmid of the other C-55PP phosphatases was well tolerated [15]. Here, we found that *lpxT* overexpression in *E. coli* blocked cell division and caused phenotypes typically associated with cell envelope damage. The IM hyper-proliferation, the altered LPS distribution in cellular fractions, and the envelope defects in the absence of L,D-transpeptidases shown by *lpxT*-overexpressing cells supported the hypothesis that *lpxT* overexpression impairs growth by interfering with LPS transport.

## 2. Materials and Methods 

### 2.1. Bacterial Strains, Plasmids, and Culture Media

Bacterial strains and plasmids are listed in Table 1. 

pLPXT^H190A^ was obtained by overlapping PCR. In brief, the wild type (wt) *lpxT* gene was amplified by PCR on BW25113 genomic DNA with FG3475 (GTGATTATTGGTCTGGTCATGC) and FG3519 (GGCTGCGCCAATCATTACTC) oligonucleotides, obtaining fragment 1, and with FG3520 (GAGTAATGATTGGCGCAGCCTGGTTTAC) and FG3473 (GGGAAGCTTGTGATACAGAAAGTTAATAAGC), giving fragment 2. The overlapping fragments 1 and 2 were used as template for PCR amplification with FG3475-FG3473. The resulting DNA fragment was digested with *Pst*I-*Hind*III and cloned in pLPXT digested with the same enzymes. pUPPS was obtained by cloning in the *Sma*I site of pGZ119HE [26], a DNA fragment obtained by PCR amplification of BW25113 genomic DNA with FG3565 (GCATGCAGGAGGATATCACCATGCGTTCGATTGCCAGA)-FG3566 (GGGGAATTCTTAACTTTCTTGTTCTCGTTGC) oligonucleotides. The gene is transcribed from *ptac* promoter, which has high basal activity also in the absence of isopropyl β-d-1-thiogalactopyranoside (IPTG) [27]. All plasmids were checked by sequencing. Liquid cultures were grown in LD broth (Bacto Tryptone, 1%; yeast extract, 0.5%; NaCl, 0.5%; pH 7–7.2) or No Salt Medium (NSM; LD without NaCl) and diluted in DIL (DIFCO Nutrient Broth, 0.1%; NaCl, 0.5%). Petri dishes were prepared with LD10 (LD broth supplemented with 1% agar). When indicated, 100 µg/mL ampicillin (amp), 30 µg/mL chloramphenicol, 0.2% glucose, and 0.01% arabinose were added to culture media. Bacterial liquid and solid cultures were always incubated at 37 °C.

### 2.2. Growth of Cultures Overexpressing lpxT

Over-night cultures were prepared by inoculating a single colony in 5 mL of LD supplemented with ampicillin and incubating the cultures for 15–16 h at 37 °C, aerated. The optical density at λ = 600 nm (OD_600_) of the culture was evaluated with the Amersham Ultrospec 2100 spectrophotometer (GE Healthcare, Little Chalfont, UK), and the over-night culture was diluted in the medium at the OD_600_ indicated in figure legends. The turbidity was analyzed at intervals during aerated incubation at 37 °C by spectrophotometric determination. Aliquots of the cultures were withdrawn, serially diluted in DIL solution, and plated on LD10 to determine viable counts. The log_10_ of either OD_600_ or bacterial titer was plotted vs. time. 

### 2.3. Flow-Cytometric Analysis 

For flow-cytometric analysis, over-night cultures of BW25113 carrying either pGM930 or pLPXT were diluted to OD_600_ = 0.01 in 10–15 mL of LD supplemented with ampicillin with or without arabinose (+ and -ARA, respectively) and incubated at 37 °C, aerated. Once the cultures reached OD_600_ = 0.5 (-ARA) or at the onset of growth arrest (+ARA), 2 mL was withdrawn and centrifuged to pellet bacterial cells. Cells were washed twice with PBS (137 mM NaCl, 2.7 mM KCl, 10 mM Na_2_HPO_4_, 1.8 mM KH_2_PO_4_; pH = 7.4) and resuspended in 1 mL of PBS, and the turbidity was adjusted to OD_600_ = 0.05. For analysis, 10^6^ gated cell events for each sample were acquired using the forward-scatter (FSC) and side-scatter (SCC) detectors in a BD (Franklin Lanes, NJ, USA) FACSCanto II flow cytometer. Data were analyzed with FlowJo software (version 10.4.2; BD, Franklin Lanes, NJ, USA).

### 2.4. Protein Analysis

Total protein extracts were prepared from bacterial cultures as described [17]. Extraction from culture media was performed by withdrawing 1.5 OD_600_ culture samples and removing the intact cells by centrifugation. The supernatants were filtered through a membrane with 0.2 µm pore size and were precipitated with trichloroacetic acid-. Protein samples were diluted 1:1 in protein loading sample buffer (50 mM Tris–HCl (pH 6.8), 2% SDS, 0.1% bromophenol blue, and 10% glycerol) and loaded onto SDS-12% polyacrylamide gels. After the run, the gels were either stained with Coomassie or with ProteoSilver Silver Stain kit (SIGMA, Saint Louis, MO, USA), or blotted onto nitrocellulose membranes and analyzed by Western blotting with anti-S1 [28] or anti-MBP PA1-989 (Thermo Fisher Scientific, Waltham, MA, USA) polyclonal antibodies. Gel fluorescence was detected with the Bio-Rad (Hercules, CA, USA) ChemiDoc MP Imaging System using the Alexa488 channel.

### 2.5. MALDI-TOF Mass Spectrometric Analyses

Cultures of BW25113/pGM930, KG-279/pGM930, and KG-279/pLPXT in LD with ampicillin were induced at OD_600_ = 0.2 with arabinose and incubated at 37 °C. Cells were collected from 3 L of culture 1 h after growth impairment for KG-279/pLPXT and at similar optical density for BW25113/pGM930 and KG-279/pGM930 (i.e., at around OD_600_ = 0.7). Cells were washed with PBS and the cell pellets were lyophilized. Dried cells for MALDI preparation were washed several times with distilled water, then with CHCl_3_/CH_3_OH (1:2, *v*/*v*) and CHCl_3_/CH_3_OH/H_2_O (3:2:0.25, *v*/*v*/*v*) to remove potential phospholipid contaminants. After these purification steps, the lipid A structure was analyzed directly on the bacterial pellet as previously described [29,30]. In parallel, an aliquot of cells from each strain underwent a small-scale extraction of the LPS by the hot-phenol water procedure [31], followed by several purification steps comprising enzymatic digestion of proteins and nucleic acids, as well as size-exclusion chromatography. An aliquot of the pure LPS of each strain underwent a mild acid hydrolysis with acetate buffer (pH 4.4, 5 h, 100 °C) to separate the lipid A portion from the saccharide part of the LPS. The solutions were extracted three times with CHCl_3_/CH_3_OH/H_2_O (100:100:30, *v/v/v*) and centrifuged as previously reported [32]. The organic phases containing the lipid A moiety were further purified by several washes with distilled water, lyophilized, and then analyzed by MALDI-TOF MS. Lipid A fractions were then dissolved in CHCl_3_/CH_3_OH (50:50, *v/v*). The matrix solution was 2,4,6-trihydroxyacetophenone in CH_3_OH/0.1% trifluoroacetic acid/CH_3_CN (7:2:1, *v*/*v*/*v*) at a concentration of 75 mg/mL. A total of 0.5 μL of the sample and 0.5 μL of the matrix solution were deposited onto a stainless steel plate and left to dry at room temperature. Each spectrum in the MS experiments was a result of the accumulation of 1500 laser shots, whereas 5000–7000 shots were summed for the MS/MS spectra. Each experiment was performed in triplicate. All the MS and the MS^2^ experiments were performed both in linear and reflectron mode, negative ion polarity on an ABSCIEX TOF/TOF 5800 Applied Biosystems (Foster City, CA, USA) mass spectrometer equipped with an Nd:YAG laser (λ = 349 nm), with a 3 ns pulse width and a repetition rate of up to 1000 Hz, and also equipped with delayed extraction technology.

### 2.6. Microscopic Observations 

For TEM analyses, samples were prepared as follows. BW25113/pGM930 and BW25113/pLPXT stationary cultures were diluted to OD_600_ = 0.01 in 25 mL of LD broth supplemented with ampicillin and arabinose. Cultures were grown at 37 °C, aerated. Then, 4 mL aliquots were taken at about OD_600_ = 0.5 for BW25113/pGM930 and 40 min after growth defect onset for BW25113/pLPXT. Samples were prepared for electron microscopy as described [33], and pictures were taken with an EFTEM Leo 912ab (Zeiss, White Plains, NY, USA) at the NoLimits platform (UNITECH, Università degli Studi di Milano, Milano, Italy).

For optical microscope analyses, samples of the culture of interest were fixed with formaldehyde (0.37% final concentration). After 30 min at 37 °C with shaking, the cells were pelleted, washed, and resuspended in PBS. A total of 5 µL of cell suspension was transferred onto a microscope slide coated with a thin layer of 1.5% agarose. 4′,6-Diamidino-2-phenylindole (DAPI) staining was performed by incubating fixed cells with 5 µg/mL DAPI for 30 min at 37°C with shaking, protected from light. Stained cells were directly transferred onto a microscope slide, as described above. Bacterial cells were observed with a Leica DMRA2 widefield microscope (Leica Microsystem, Wetzlar, Germany) using the 100x immersion objective. DAPI and superfolder Green Fluorescent protein (sfGFP ) signals were visualized in the UV or fluorescein isothiocyanate channels, respectively. The length of the cells was measured with ImageJ Software.

### 2.7. Fluorescence Determination

Two milliliter samples were taken from cultures producing fluorescent proteins and were centrifuged to collect bacterial cells. The pellets were resuspended in cold 0.9% NaCl solution at OD_600_ = 0.5 and incubated in ice around 1 h. Fluorescence_485/535_ (F_485/535_) and OD_600_ were measured by means of an Ensight (PerkinElmer, Waltham, MA, USA) microplate reader. Green fluorescent protein (GFP) activity (F_485/535_/OD_600_) was expressed in arbitrary units (AU). 

### 2.8. Sucrose Gradient Fractionation

IMs and OMs were fractionated as described [34]. BW25113/pLPXT over-night cultures were diluted to OD_600_ = 0.05 in 500 mL of LD supplemented with ampicillin and incubated at 37 °C, aerated. At OD_600_ = 0.2, cultures were induced or not with 0.01% arabinose. A total of 250 OD were collected from culture (4000× *g*, 15 min, 4 °C) 90 min after the induction and at similar optical density for the uninduced culture (i.e., at around OD_600_ = 0.5–0.6). Cells were resuspended in 4 mL of 10 mM Tris-HCl, pH 7.8, containing 20% sucrose (*w/w*), 1 mM phenylmethylsulfonyl fluoride (PMSF), and 50 μg/mL DNase I, and lysed by a single passage through a Cell Disruptor (One Shot Model by Constant Systems LTD) at a pressure of 8000 psi. Unbroken cells were removed by centrifugation (4000× *g,* 10 min, 4 °C). A total of 1.6 mL of cell lysate was layered onto the 10 mM Tris-HCl (pH 7.8)-containing sucrose gradient solutions (*w/w*), stratified from the top as follows: 30% (0.45 mL), 35% (1.26 mL), 40% (2.16 mL), 45% (1.26 mL), 50% (1.26 mL), 55% (0.9 mL), 60% (0.45 mL). Samples were ultracentrifuged at 180,000× *g* for 16 h (SW41Ti rotor, OPTIMA XL-90 Beckman Coulter, Brea, CA, USA). Then, 0.4 mL fractions were collected from the top. Odd fractions were mixed with 5X sample loading buffer (250 mM Tris– HCl, pH 6.8; 5% SDS; 50% glycerol; 0.25% bromophenol blue; 5% β-mercaptoethanol) and loaded onto SDS-12% polyacrylamide gels. Gels were either stained with Coomassie or blotted onto nitrocellulose membranes and analyzed by Western blotting with anti-LptC (kindly provided by A. Polissi) or anti-MBP PA1-989 (Thermo Fisher Scientific, Waltham, MA, USA) polyclonal antibodies. For LPS visualization, samples were loaded onto Tricine-SDS-18% gels [35] and immunodetected using anti-LPS core WN1 222-5 monoclonal antibodies (HyCult Biotechnology, Wayne, PA, USA). 

## 3. Results

### 3.1. lpxT Overexpression Had a Bacteriostatic Effect on E. coli Growth 

**Figure 2:**
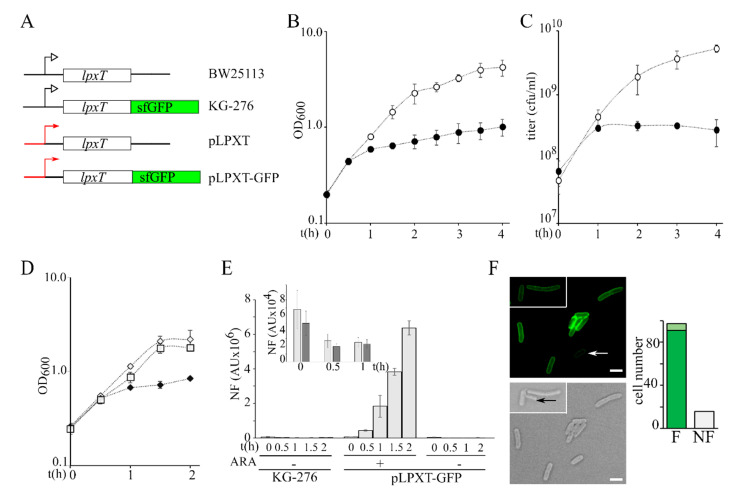
Growth of *lpxT*-overexpressing cultures and analysis of LpxT_sfGFP_ protein expression. (**A**) Scheme of *lpxT* locus in BW25113 and in chromosomal (KG-276) and plasmid (pLPXT and pLPXT-GFP) constructs [17] (not drawn on scale). Black lines represent chromosomal regions flanking the *lpxT* gene. Red lines and arrows represent the *araBp* promoter. Black bent arrows represent the *lpxTp* promoter. (**B**,**C**) Exponential BW25113/pLPXT cultures at OD_600_ = 0.2 were split in two and supplemented (filled symbols) or not (empty symbols) with 0.01% arabinose. Growth at 37 °C was followed by reading the OD_600_ (**B**) or by plating serial dilution on LD10 plates with ampicillin (**C**). Average results of at least three independent replicate experiments are reported with standard deviation. (**D**) Exponential BW25113/pLPXT-GFP cultures at OD_600_ = 0.2 were split in two and supplemented (filled diamonds) or not (empty diamonds) with 0.01% arabinose. The growth curve of KG-276 is also reported (empty squares). Average results of three independent replicate experiments are reported with standard deviation. (**E**) Fluorescence was measured on samples taken at the time indicated under the columns from cultures growing as shown in (**D**). In the box, fluorescence of KG-276 (light grey bars) and of uninduced BW25113/pLPXT-GFP (dark grey bars) cultures is shown. In all cases, the columns represent the average of three determinations with standard deviation. N.F., normalized fluorescence. AU, arbitrary units. (**F**). Induced BW25113/pLPXT-GFP cells imaged at the fluorescence microscope. Scale bar, 2.5 µm. White arrow, cell with low fluorescence; black arrow, cell not fluorescent. The columns in the histogram represent the number of cells out of 113 showing high (**F**, dark green), low (**F**, light green), or no fluorescence (NF) 1 h after the induction.

To evaluate the effect of *lpxT* overexpression, we cloned the *lpxT* gene into plasmid pGM930 [25] under the transcriptional control of the *araBp* promoter, inducible with arabinose (plasmid pLPXT, Figure 2A) [17], and analyzed the growth of *E. coli* BW25113/pLPXT upon *lpxT* induction.

We performed all the experiments by inducing *lpxT* with 0.01% arabinose, a condition that does not affect the growth of BW25113/pGM930 control strain (Figure S1A). We observed that the growth rate of BW25113/pLPXT exponential cultures did not change for the first 30 min post-induction (p.i.) and then strongly decreased (Figure 2B). To check whether LpxT had a bacteriostatic or bactericidal effect on growth, we titered the cultures carrying pLPXT and we found that the titer increased for the first hour after arabinose addition and then it started slowly decreasing (Figure 2C), passing from 3.2 × 10^8^ at 1 h p.i. to 5 × 10^7^ colony forming units (cfu)/mL at 24 h p.i. Notably, in the same timespan, the optical density of *lpxT*-overexpressing cultures increased almost fivefold (from 0.6 ± 0.03 to 2.9 ± 0.4), suggesting that suppressors and/or cells that had lost pLPXT had accumulated over time. To test the hypothesis of pLPXT plasmid instability, we titered overnight BW25113/pLPXT cultures grown in LD with arabinose on plates containing or not containing ampicillin. We found that the efficiency of plating (eop) was around 50-fold lower on plates with ampicillin with respect to plates without the antibiotic, suggesting that less than 2% of the cells contained pLPXT. Conversely, no difference was found in the eop of BW25113/pGM930 or uninduced BW25113/pLPXT cultures in the presence or absence of the antibiotic (Table 2 and Figure S1B).

To estimate the amount of LpxT that inhibited growth, we exploited a pLPXT derivative with an *lpxT*-sfGFP translational fusion under the *araBp* promoter (plasmid pLPXT-GFP; Figure 2A) [17], which allowed us to quantitate the LpxT_sfGFP_ fusion protein as fluorescence intensity. LpxT_sfGFP_ was previously shown to be correctly targeted to the inner membrane [17] (see also Figure 2F). The induction of *lpxT*-GFP, like that of *lpxT*, caused growth inhibition between 30 and 60 min p.i. (Figure 2D). In this timespan, the fluorescence intensity of the induced BW25113/pLPXT-GFP cultures was between around 6- and 25-fold higher than that of the KG-276 strain (Figure 2E), i.e., a BW25113 derivative with the *lpxT*-sfGFP fusion integrated at the *lpxT* chromosomal locus (Figure 2A). On the other hand, the uninduced cultures had a fluorescence similar to that of KG-276 (Figure 2E, box). We also examined cells carrying pLPXT-GFP by fluorescence microscopy 1 h p.i. to check the intracellular distribution of the protein and to assess whether the population of induced cells had homogenous fluorescence intensity. Fluorescence was evenly distributed at the cell periphery, as expected for a membrane protein. A total of 85% of the cells were fluorescent, with a major fraction of cells showing similar fluorescence intensity and a small fraction showing faintly fluorescent cells (Figure 2F).

### 3.2. lpxT Overexpression Interfered with Cell Division 

**Figure 3:**
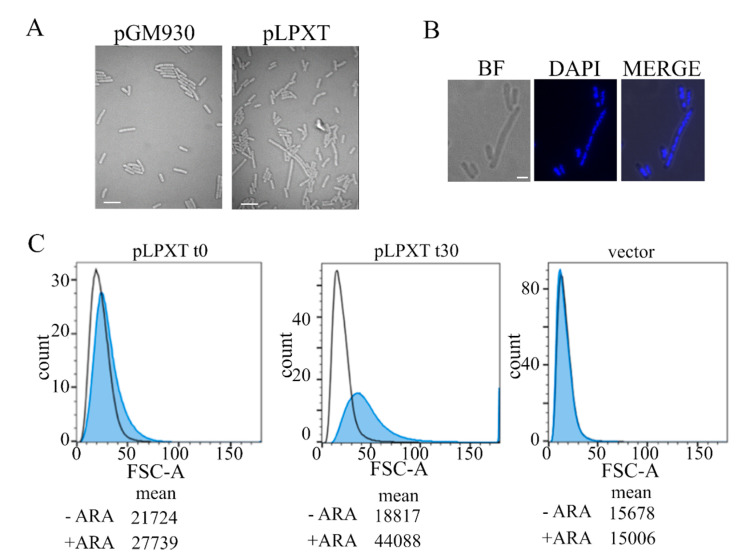
Microscopy and flow cytometry analyses of cell morphology. (**A**) Cultures of BW25113 carrying pLPXT or pGM930 (vector) were grown and induced with arabinose, as described in the legend of Figure 2B. Samples of BW25113/pLPXT cultures were withdrawn 60 min after growth arrest or at a comparable OD_600_ (i.e., around 0.6) for BW25113/pGM930 cultures. Scale bars, 3 µm. (**B**) Induced BW25113/pLPXT cells stained with 4′,6-diamidino-2-phenylindole (DAPI) and observed by bright field (BF) or UV (DAPI) microscopy. The two images were merged with ImageJ (MERGE). Scale bar, 1 µm. (**C**) Samples were taken from the control cultures (i.e., -ARA, uninduced BW25113/pLPXT; vector, BW25113/pGM930) at OD_600_ = 0.5 and from induced BW25113/pLPXT cultures (+ARA) immediately after growth arrest (t0) or 30 min later (t30). Histogram of forward scatter area for BW25113 cells carrying pLPXT or pGM930 plasmids, uninduced (black line) or induced with ARA (blue-filled peaks). Geometric means of the forward scatter area are reported in arbitrary units (AU). Flow cytometer data are representative of two independent experiments.

Microscopic observation of *lpxT*-overexpressing cells showed that induced cells were larger than normal and heterogeneous in length, with some of them forming chains or filaments (Figure 3A). The elongated cells often contained segregated nucleoids (Figure 3B), suggesting that DNA replication and segregation were not impaired.

Flow cytometry of samples taken from cultures of BW25113/pLPXT at two successive time points after *lpxT* induction (Figure 3C) showed that cells overexpressing *lpxT* were slightly larger than the uninduced ones or those carrying the pGM930 vector at the time of growth arrest. Then, 30 min later, the distribution of induced cells was wider and rightward shifted, with a separated peak of cells with a forward scatter area much larger than the others, in agreement with generally enhanced cell size and filamentation of some cells.

**Figure 4:**
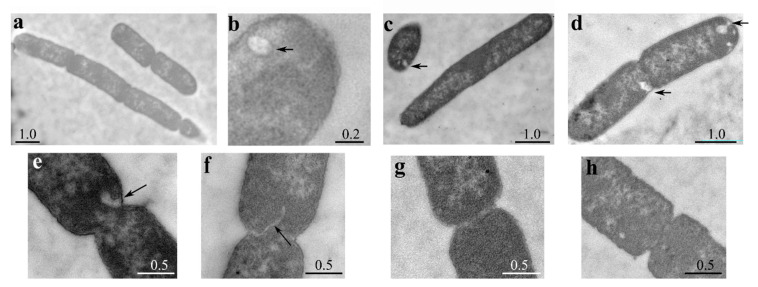
Ultrastructural cell analysis by TEM. Cultures of BW25113 carrying pLPXT (**a**–**f**) or pGM930 (**g**,**h**) were inoculated in LD with ampicillin and 0.01% arabinose. Samples were taken 30 min after the growth arrest for BW25113/pLPXT or at OD_600_ = 0.5 for the control strain and prepared for TEM observation. Arrows indicate membrane invaginations or vesicles. Scale bars (in µm) are shown on the panels. Frequency of BW25113/pLPXT cells with altered septa was significantly higher than that of BW25113/pGM930 cells (13 out of 17 vs. 4 out of 13 cells with visible constrictions; *p* = 0.012 evaluated with Pearson’s chi-squared test).

To better characterize the morphological defects associated with LpxT excess, we examined the cellular ultrastructure by transmission electron microscopy (TEM). As shown in Figure 4, we observed both chains of 3–4 unsegregated cells (a) and elongated cells without visible constrictions, although in some cases with segregated nucleoids (c). When constrictions were present, septa were in most cases either not visible or altered (e,f) with respect to those observed in the strain carrying the empty vector (g,h). Moreover, IM invaginations were frequently observed (c,d,e), and intracellular vesicles were also present in some cells (b). Overall, microscopy data showed that excess LpxT impaired cell division, probably at the level of septum formation, and caused IM proliferation. 

### 3.3. lpxT Overexpression Enhanced Cell Permeability and Confers Sensitivity to Hypo-Osmotic Stress

**Figure 5:**
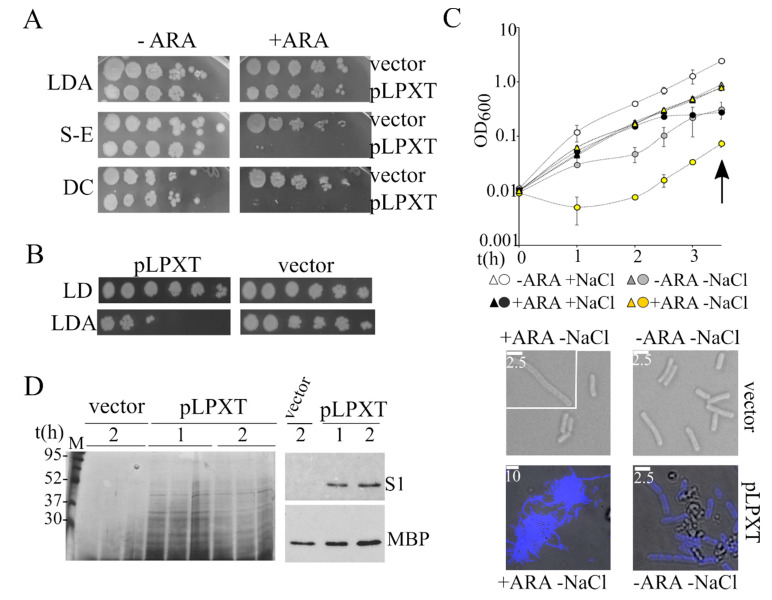
Analysis of the *lpxT*-overexpressing strain permeability and sensitivity to osmotic shock. (**A**) Cultures of BW25113 carrying either pGM930 (vector) or pLPXT (pLPXT) were grown 16 h at 37 °C in LD with ampicillin and serially diluted (x10) in a 96-well plate. Dilutions were replicated on LD10 with ampicillin 100 μg/mL (LDA) with (+ ARA) or without (−ARA) 0.2% arabinose and incubated for around 20 h at 37 °C. S-E and DC, plates supplemented with ampicillin and either 0.5% SDS-0.8 mM ethylenediaminetetraacetic acid (EDTA) (S-E) or 1% sodium deoxycholate (DC). (**B**) Single colonies obtained in diluted spots plated on LDA with arabinose (panel A) were touched with a toothpick and resuspended in 0.1 mL of LD in a 96-well plate. The suspension was serially diluted (×10) and plated on LD10 with or without ampicillin. The experiment was repeated on eight independent colonies for each strain with similar results. (**C**) Exponential BW25113/pLPXT (circles) and BW25113/pGM930 (triangles) cultures growing in LD with ampicillin and arabinose were diluted to OD_600_ = 0.01 in either LD (+NaCl) or No Salt Medium (NSM; −NaCl) in the presence (+ARA) of arabinose and ampicillin. Control uninduced cultures were similarly diluted in media without arabinose (−ARA). The cultures were incubated at 37 °C, measuring the optical density at intervals. Average results relative to three independent cultures for each strain/condition are reported with standard deviation (upper part; in some cases, error bars are hidden by the symbols). At the time indicated by the arrow, samples were withdrawn, and the cells were observed by the microscope (lower part). To better distinguish cells carrying pLPXT from debris, the cells were stained with DAPI and bright field, and fluorescence microscopy images were merged with ImageJ. White lines, scale bars in micrometers. (**D**) Analysis of proteins released in culture medium. Cultures of BW25113 carrying pGM930 or pLPXT were grown in LD with ampicillin and arabinose. Samples were taken at the time of growth arrest of the induced BW25113/pLPXT culture (1) and 30 min later (2), treated as described in the Materials and Methods section and run in a 12% polyacrylamide gel. Gels were either silver stained (left panel) or blotted and immunodecorated with antibodies specific for S1 ribosomal protein or MBP maltose binding periplasmic protein (right panel). The results relative to three independent cultures of the two strains are shown in the left panel.

BW25113/pLPXT cultures had similar eop on ampicillin plates containing or not containing arabinose (Figure 5A). However, the colonies obtained in the presence of arabinose were mainly composed of ampicillin-sensitive cells, suggesting that during growth on solid medium, the cells readily lost the pLPXT plasmid (Figure 5B). Conversely, BW25113/pLPXT growth on arabinose plates was strongly inhibited by the addition of either SDS-EDTA or sodium deoxycholate (Figure 5A), implying that *lpxT* overexpression weakened the cell envelope permeability barrier. 

We wondered whether ectopically expressed LpxT may also cause hypersensitivity to hypo-osmotic stress, a phenotype associated with cell envelope defects [36,37]. To test this hypothesis, we diluted cultures of BW25113/pLPXT arrested by *lpxT* induction in LD or LD without NaCl (NSM medium), both containing arabinose. In parallel, we analyzed uninduced BW25113/pLPXT cultures diluted in LD and NSM without arabinose and control cultures carrying the pGM930 vector. As shown in Figure 5C, salt and/or arabinose presence did not affect the growth of the control strain; accordingly, the cell morphology of cells adapted to NSM was normal, with a mean length of 3.8 µm and rare elongated cells both in presence and absence of arabinose. 

Conversely, the turbidity of induced BW25113/pLPXT cultures diluted in NSM with arabinose showed an initial drop and started increasing after a roughly 1 h lag. Even without the inducer, the cultures grew slowly for the first 1–2 h upon dilution in NSM, showing that LpxT basal expression from the plasmid was sufficient to increase cell sensitivity to hypo-osmotic shock. 

Microscopic observations showed that BW25113/pLPXT formed large aggregates of filamentous cells when growing in NSM with arabinose. Smaller aggregates and cellular debris were observed also in uninduced BW25113/pLPXT cultures (Figure 5C), whereas they were not found in BW25113/pGM930 cultures or in BW25113/pLPXT cultures growing in LD broth. 

To test whether LpxT can make the cells prone to lyse also in LD, we analyzed the proteins released in the spent medium of BW25113/pLPXT at the time of growth arrest (i.e., 1 h p.i.) and 1 hour later. As shown in Figure 5D, we found heterogeneous proteins in the spent medium of cultures overexpressing *lpxT* and not in that of BW25113/pGM930 control cultures. The overall protein amount at the two time points did not increase, suggesting that they had been released during growth. Western blotting with antibodies specific for either the periplasmic protein MBP or the cytoplasmic ribosomal protein S1 showed that MBP was released in the medium by both strains, albeit in a lower amount by cells carrying the pGM930 vector than by those with pLPXT, whereas S1 was present only in the medium recovered from cultures of *lpxT*-overexpressing cells. This result was consistent with the idea that LpxT overproduction may cause cell envelope damage, making the cells more prone to lyse. 

### 3.4. lpxT-Dependent Growth Arrest Was Relieved by Glucose 

Upon dilution in LD, cultures arrested because of *lpxT* overexpression started to grow without a significant lag, irrespective of the presence of arabinose (Figure S2A). We therefore hypothesized that the glucose present in LD (between 10 and 40 µM in different batches [38]) could be sufficient to switch off the *araBp* promoter, which is sensitive to catabolite repression [27], until the cells were at low density and did not consume it completely, leading to a transient decrease in LpxT amount. In agreement with this hypothesis, 30 min after the dilution in LD with either glucose or arabinose of BW25113/pLPXT-GFP-induced cultures, the amount of LpxT-GFP protein was similarly reduced (Figure S2A). 

### 3.5. UppS Did Not Suppress LpxT-Dependent Growth Defect 

Considering that LpxT transfers a phosphate group from C55-PP to the LPS molecule, its excess may potentially interfere with C55-PP recycling and/or LPS transport and modification status, impairing in turn the PG and the OM integrity, respectively. Indeed, hindering C55-PP recycling prevents growth and causes cell morphology defects that can be suppressed by increased production of C55-PP [15,39]. However, as shown in Figure S2B, ectopic expression of the *uppS* C55-PP synthase gene had no effect on the growth of the strain overproducing LpxT, arguing against the hypothesis that LpxT may impair the C55-PP recycling pathway. 

### 3.6. lpxT Overexpressing Cells Have Features Suggestive of Defective LPS Transport 

**Figure 6:**
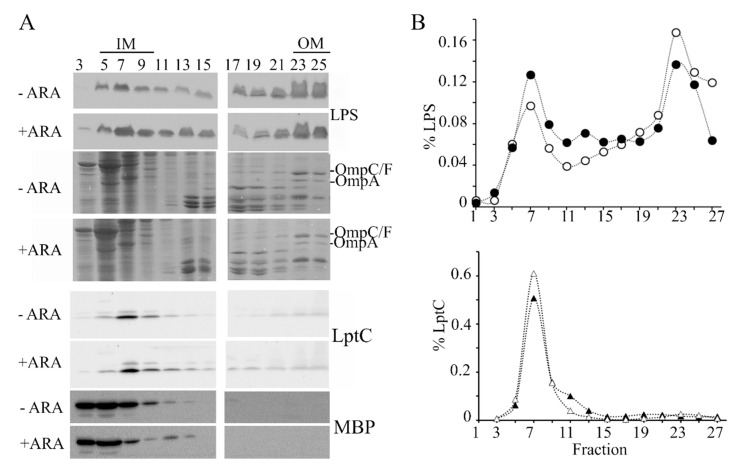
Distribution of LPS in sucrose gradient fractions. Cultures of BW25113/pLPXT were grown in LD with ampicillin up to OD_600_ = 0.2 and supplemented or without 0.01% arabinose. Cells were collected 90 min after the induction (+ ARA) and at similar optical density for the uninduced (-ARA) culture (i.e., at around OD_600_ = 0.5–0.6). IMs and OMs were separated by isopycnic sucrose gradient centrifugation as described in the Materials and Methods section. (**A**) LPS distribution across fractions was determined by Tricine-SDS-PAGE and immunoblotting using anti-LPS WN1 222-5 antibody. The profiles of the major OM porins (OmpC/F, OmpA) were determined by SDS-PAGE followed by Coomassie staining. IM and periplasmic fractions were identified by SDS-PAGE and immunoblotting using antibodies specific for LptC IM protein or MBP periplasmic protein. (**B**) LPS and LptC signals of induced (filled symbols) or uninduced (empty symbols) samples were quantified by densitometry with Image Lab (Biorad, Hercules, CA, USA) and normalized for the total LPS or LptC signals. LPS distribution in fractions 1–19 vs. 21–27 was significantly different between induced and uninduced cells according to Pearson’s chi-squared test (*p* < 0.0001).

To explore whether excessive LpxT hampered LPS transport, we analyzed the cellular distribution of LPS by sucrose gradient fractionation of cell extracts. In fact, in cells defective in the LPS transport because of the depletion of some Lpt components, the LPS accumulated in fractions with intermediate density between that of the IM and the OM (light and heavy fractions, respectively). Moreover, an anomalous form of LPS decorated with colanic acid is produced in such strains [40,41]. We observed that the LPS amount associated with light and intermediate fractions (fractions 1–19) with respect to that in the heavy fractions (fractions 21–27) was higher in *lpxT*-overexpressing cells than in the uninduced cultures (Figure 6A,B; see also the results of a replicate experiment in Figure S3), thus suggesting that the LPS transport from the IM to the OM was actually less efficient in such cells. On the other hand, LPS forms with altered migration were not detected.

**Figure 7:**
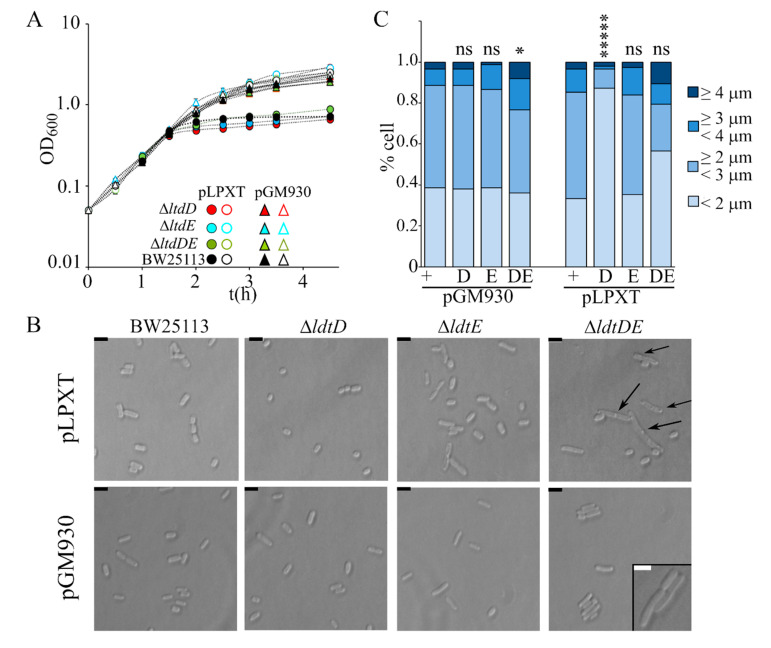
Effect of *lpxT* overexpression in strains lacking LdtD and/or LdtE. Growth curve (**A**) and cell morphology (**B**,**C**) of BW25113 (+), AMM05 (D, Δ*ldtD*), AMM06 (E, Δ*ldtE*), and AMM07 (DE, Δ*ldtDE*) carrying either pGM930 or pLPXT, as indicated in the panels. (A) Cultures were grown at 37 °C with aeration in LD with ampicillin up to OD_600_ = 0.2, split in two, and supplemented (filled symbols) or not (empty symbols) with 0.01% arabinose. Growth at 37 °C was followed by reading the OD_600_. For all mutant strains, the results are the average of three independent replicate experiments. Error bars (in most cases hidden by symbols) refer to standard deviation. The growth curves of BW252113 carrying either pGM930 or pLPXT are reported for comparison. (**B**,**C**) Cells of the indicated strains were observed at the optical microscope after growth 16 h at 37 °C with aeration in LD with ampicillin and 0.01% arabinose. (B) The arrows point to visible knobs on the cells. Scale bars, 2.5 μm; box scale bar, 2 μm. (C) Percentage of cells with different length range (*n* = 150; measured with ImageJ). *t*-test results (with respect to the BW25113 carrying the same plasmid, + columns) are shown on top of the bars. ns, not significant; *, *p* < 0.05; *****, *p* < 0.00001.

In a recent paper, it was reported that cells with OM stress due to inefficient LPS transport lyse in the absence of the L,D-transpeptidase LdtD and Ltd.E [23,42], suggesting that PG remodeling may partially compensate OM defects. We thus analyzed the effect of single Δ*ldtD*, Δ*ldtE*, and double Δ*ldtDE* mutations on the growth and morphology of *lpxT*-overexpressing cells. All strains stopped growing around 60 min after arabinose addition, such as the wt (Figure 7A). Microscopic observations showed that Δ*ldtD* and Δ*ldtE* cells were similar to those of the BW25113 strain, whereas Δ*ldtDE*/pLPXT cells occasionally showed small knobs (data not shown). After long incubation with arabinose, the turbidity reached by Δ*ldtD* and, to a lesser extent, Δ*ldtE* cultures was reduced, although the titer was not significantly different with respect to that of BW25113 cultures (Table 2). Microscopic observations showed that Δ*ldtD*/pLPXT cells were very small and with a rounded morphology with only rare long cells (Figure 7B,C), thus explaining the low turbidity of the cultures. Moreover, Δ*ldtDE*/pLPXT elongated cells had almost invariably evident knobs (Figure 7B), which were also present in the rare elongated Δ*ldtD*/pLPXT cells (data not shown) and absent in the mutant strains carrying pGM930 (Figure 7B), suggesting that LpxT may cause cell envelope defects that can be counteracted by L,D-transpeptidase activity.

### 3.7. Lipopolysaccharide Lipid A Phosphorylation Level Was Not Altered in lpxT-Overexpressing Cells 

**Figure 8:**
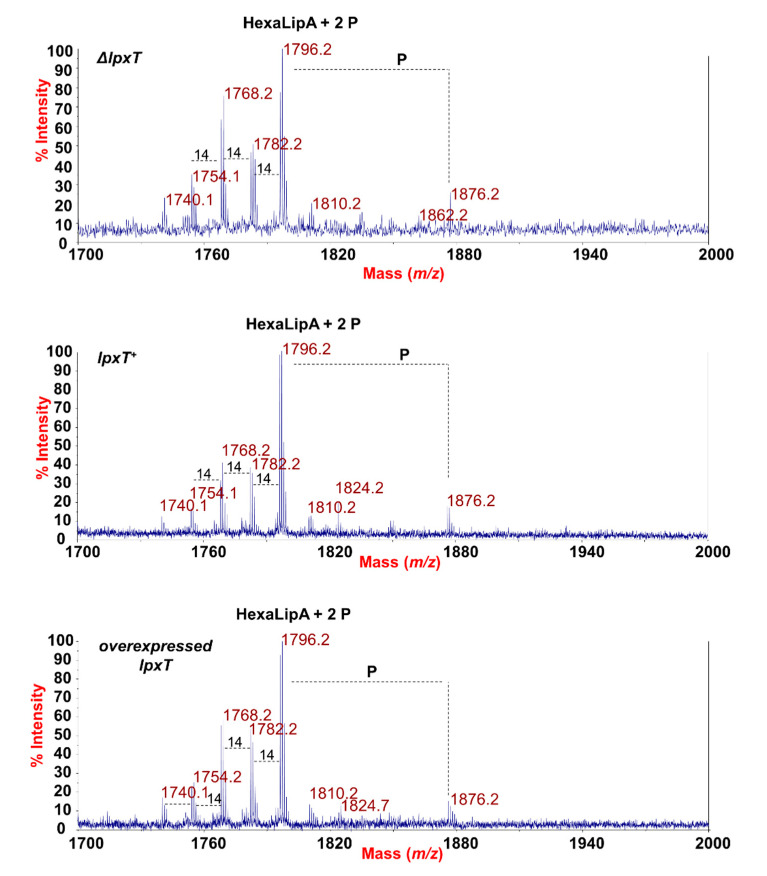
MALDI-TOF mass spectrometry analysis of lipid A. Negative-ion MALDI-TOF mass spectra of intact bacterial cultures of KG-279/pGM930 (Δ*lpxT*), BW25113/pGM930 (*lpxT*^+^), and KG-279/pLPXT (*lpxT* overexpressed) grown as described in the Materials and Methods section. Assignment of the lipid A species as HexaLipA (hexa-acylated lipid A species) and relative group of ions further decorated by a third phosphate is indicated.

In order to evaluate whether the overexpression of LpxT could affect the structure of the LPS, we analyzed the structure of the lipid A from *lpxT*^+^, Δ*lpxT*, and *lpxT*-overexpressing strains by employing two parallel mass spectrometry (MS)-based approaches. Phosphates (i.e., phosphodiester bonds) are labile decorations that could be lost with the typical chemical treatment used to isolate the lipid A from the remaining saccharide portion of an LPS. Therefore, MS analysis directly on the intact bacterial cells was executed as a first approach [29,30]. In parallel, the lipid A structures were also analyzed following mild acid hydrolysis of the extracted and purified LPSs [43,44]. MS investigations performed on the intact cells (Figure 8) revealed no relevant differences among the strains in the lipid A species profile, with the prevalence of a mixture of hexa-acylated lipid A forms decorated by two phosphates for all the strains analyzed. 

**Figure 9:**
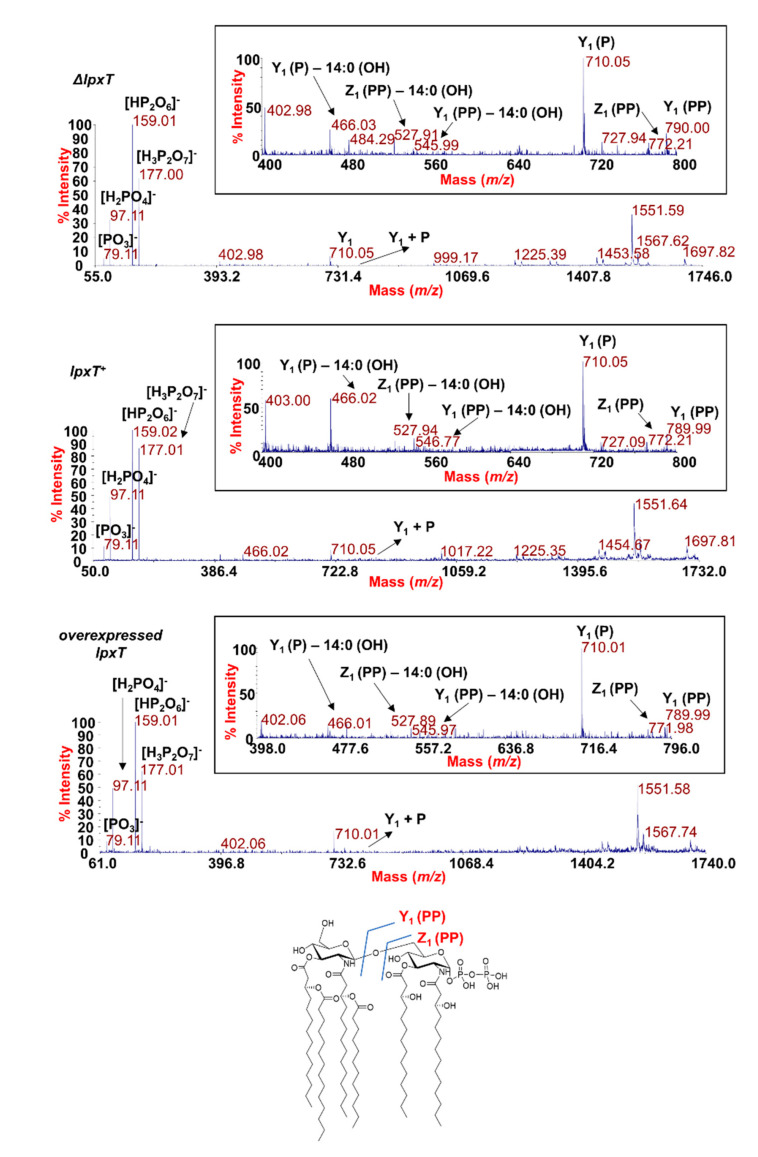
MALDI-TOF MS^2^ analysis of hexa-acylated lipid A species carrying two phosphate units. Negative-ion MALDI MS^2^ spectra of precursor ions at *m/z* 1796.5 of the lipid A isolated from KG-279/pGM930 (Δ*lpxT*), BW25113/pGM930 (*lpxT*^+^), and KG-279/pLPXT (*lpxT* overexpressed). This is a representative ion peak of the cluster ascribed to hexa-acylated lipid A species decorated by two phosphates. A zoom of the *m/z* 400–800 region with the key fragments’ assignment is reported for each spectrum. The structure of a representative hexa-acylated lipid A species illustrating the key product ions derived from the glycosidic bond cleavage that allowed the location of the two phosphate groups as a pyrophosphate decorating the reducing glucosamine unit is reported.

The main species was detected at *m/z* 1796.2 (Figure 8) matching with the typical *E. coli* lipid A glucosamine disaccharide backbone carrying four 14:0 (3-OH), one 14:0, and one 12:0 acyl chains and decorated by two phosphates. Moreover, peaks with mass differences of 28 (-CH_2_CH_2_- unit) and 14 (-CH_2_- unit) amu (atomic mass unit), indicative of the occurrence of species differing in the length of the acyl chains, were also found, as previously reported for other *E. coli* strains [45]. Importantly, a peak at *m/z* 1876.2 corresponding to *tris*-phosphorylated lipid A species (i.e., carrying a pyrophosphate group) was observed in all the strains (Figure 8). Negative-ion MS analysis of the isolated lipid As (Figure S4 and Table S1) showed the occurrence mainly of heterogenous hexa-acylated species, but also of penta- and tetra-acylated forms decorated by two phosphate groups. Mono-phosphorylated lipid A species at *m/z* 1716.6, 1490.3, and 1280.1 were also detected (Figure S4 and Table S1). On the other hand, it should be noted that, in the case of MS analysis of lipid A after mild acid hydrolysis of LPSs, no species decorated by three phosphates were identified, probably due to the isolation procedure. The analysis of negative-ion MALDI MS^2^ spectra of precursor ions at *m/z* 1796.5 (Figure 9) of the mild acid hydrolysis products revealed that, consistent with what previously reported [46], the lipid A of all strains, including the Δ*lpxT*, consisted of a heterogenous mixture of pyrophosphorylated and *bis*-phosphorylated species.

To define the exact location of the two phosphate groups, a detailed analysis of each product ion was performed. Despite in all the MS^2^ spectra the most abundant product ions (*m/z* 159 and *m/z* 177) were assignable to pyrophosphate-derived ions, we cannot consider these as indicative of the actual presence of pyrophosphorylated lipid A species (43); rather, the actual occurrence of lipid A species carrying a pyrophosphate group on the reducing glucosamine unit was proven by the observation of key product ions derived from glycosidic bond cleavages where the resulting anions contained two phosphate groups. These products ions were Z_1_ (PP) ion at *m/z* 772.2 and Y_1_ (PP) ion at *m/z* 789.9, in addition to their relative product ions void of one 14:0 (3-OH) acyl chain (see also the inset of each spectrum in Figure 9) [47]. 

The above MS data show that the amount of *tris*-phosphorylated lipid A was not higher than normal in cultures blocked by excessive LpxT, suggesting that LpxT catalytic activity may be dispensable for growth arrest. To test this hypothesis, we assayed the effect on cell growth and viability of the overproduction of an LpxT variant in which His-190, a conserved residue located in the predicted active site [16], was replaced with an alanine (H190A). LpxT^H190A^ was previously demonstrated to be unable to both complement the Δ*lpxT* mutation and compete with EptA for the modification of the 1-phosphate group of lipid A [19]. We found that induction of the mutated *lpxT*^H190A^ allele tightly inhibited the growth in liquid cultures (Figure S5A) and the mutant formed colonies smaller than those of the strain overexpressing the wt allele (mainly composed by ampicillin-sensitive cells like for the wt). This effect was particularly evident in plates lacking NaCl (Figure S5B). Thus, the *lpxT*^H190A^ mutation did not abolish growth inhibition and made the protein more detrimental for growth than the wt LpxT. 

## 4. Discussion

Ectopic expression of different inner membrane proteins (IMP) is toxic to *E. coli*. This is a phenotype that has been ascribed either to generic effects, such as perturbations of IM integrity due to excessive protein insertion or stuffing of protein transport systems, or to the specific function and biochemical properties of the overexpressed IMPs [48,49]. In this work, we characterized the effects of an excessive production of the membrane protein LpxT. Although it is possible that, upon long induction, LpxT accumulation may lead to protein aggregation or irreversible IM damage, the overall phenotypes shown by *lpxT*-overexpressing cells suggest that specific effects ascribable to LpxT activity may cause the protein toxicity.

Interestingly, *lpxT*-overexpressing cells are more prone to lyse and have altered morphology, as they become progressively longer than normal and septa are mostly absent or altered even in presence of constrictions and/or segregated nucleoids, suggesting that LpxT may impair septum biosynthesis. Moreover, *lpxT*-overexpressing cells are sensitive to detergents and to hypo-osmotic shock. Such phenotypes can be explained in terms of a defective OM and/or PG biosynthesis. In fact, although decreased resistance to turgor pressure has traditionally been linked to PG defects, different studies have demonstrated that the OM is pivotal also in the mechanical protection of the cells [36,37]. Another phenotype of *lpxT*-overexpressing cells that can be a consequence of a defective cell envelope is the auto-aggregation in non-salt medium. In fact, auto-aggregation could be stimulated by altered properties of the cell surface, such as increased hydrophobicity, or by the production of exopolysaccharides and specific proteins [24,50,51]. Genes of the Cpx and Rcs regulons controlling capsular synthesis (and biofilm formation) are activated in response to envelope stress [52] and, indeed, mutants with severe defects in the LPS core biosynthesis overproduce the exopolysaccharide colanic acid in hypotonic medium, probably to compensate the loss of OM integrity and counteract the turgor pressure [53]. 

As LpxT function requires the interaction with both C55-PP and lipid A, its dysregulated expression could actually affect the transport of PG precursors and/or lipid A. It has been reported that sequestering C55-PP in dead-end intermediates of enterobacterial common antigen (ECA) biosynthetic pathway causes cell filamentation that is suppressed by increased C55-PP synthesis [39]. However, as overexpressing LpxT should actually increase the Und-PP recycling, it is unlikely that it could exert a similar sequestering effect. Accordingly, the expression of the UppS C55-PP synthase gene from a multicopy plasmid does not relieve the phenotype due to *lpxT* overexpression. Although it is possible that LpxT may interact with factor(s) involved in PG biosynthesis, as in the case of UppP and PgpB phosphatases forming a complex with Pbp1B [54], we strongly believe that overproduced LpxT may interfere with lipid A transport because several phenotypic traits of *lpxT*-overexpressing cells are reminiscent of cells with defects in the lipid A transport machinery. 

Indeed, the lack of a functional MsbA flippase causes growth inhibition, lipid A accumulation in the IM with appearance of vesicle- or tubule-like shapes, and membrane stacks. Moreover, MsbA-depleted cells show enlarged size [55,56]. In addition, Lpt complex depletion has a bacteriostatic effect accompanied by moderate lysis and formation of short filaments. Moreover, IM hyper-proliferation with formation of vesicles containing membrane material, as well as accumulation of newly synthesized LPS in the IM and other cellular fractions less dense than the OM, have been observed in Lpt-depleted cells [23,40,41,42,57]. All the above phenotypes are visible in *lpxT*-overexpressing cells. Nevertheless, IM proliferation seems less severe than in MsbA- or Lpt-depleted cells, and the lack of L,D-transpeptidases is better tolerated by *lpxT*-overexpressing cells than by the Lpt-depleted cells [23]. Differently from Lpt-depleted cells [41], *lpxT* overexpressing cells do not accumulate LPS species decorated with colanic acid, suggesting that lipid A sequestering by LpxT may prevent not only its transport, but also its modification by other enzymes. It would be interesting to assay whether LpxT excess may also prevent O-antigen ligation in *E. coli* strains that, differently from *E. coli* K12 [8], synthesize a complete LPS.

It has been reported that *E. coli* cells growing in rich medium at 37 °C contain about 750, 1000, and 500 copies of LpxT, MsbA, and LptC, respectively [58]. On the other hand, lipid A is synthesized and translocated to the outer membrane at an estimated rate of 70,000 molecules per min [59]. Thus, it seems unlikely that an interaction between LpxT and the lipid A may affect lipid A transport unless postulating that LpxT excess in proximity of the Lpt complex and/or the MsbA flippase interferes with the activity of the transporters by hindering the lipid A flow either from MsbA to the Lpt complex or within the Lpt machinery (Figure 1). However, we cannot rule out that local IM damage caused by excessive LpxT insertion may in turn impact the activity of the LPS transporters.

After prolonged incubation in inducing conditions of cells carrying pLPXT plasmid, the titer of ampicillin-resistant cells decreases, and ampicillin-sensitive cells accumulate, implying that only the cells that have lost (or rearranged) pLPXT can grow, whereas the others do not divide and (in part) die. This occurs also when uninduced cultures are plated on solid medium with arabinose, as single colonies obtained in such conditions are mostly composed by ampicillin-sensitive cells. It is possible that upon *lpxT* induction, beta-lactamase activity of plasmid-bearing cells lowers the concentration of ampicillin, thus facilitating the growth of bacteria that have lost the toxic pLPXT plasmid. 

Finally, we observed that *tris*-phosphorylated lipid A was not enhanced in *lpxT* overexpressing cells, as shown by MALDI-TOF MS, probably because of feedback regulation mechanisms that limit LpxT activity when the *lpxT* gene is induced [17,18,21]. This result rules against the hypothesis that LpxT toxicity may be due to hyperphosphorylation of lipid A. Contextually, an LpxT^H190A^ variant devoid of catalytic activity [19] is even more detrimental for growth, at least in solid medium, than the wt protein. By our MS dual approach, we reproducibly detected the presence of a peak corresponding to *tris*-phosphorylated lipid A also in the Δ*lpxT* strain. Consistent with this result, negative-ion MALDI MS^2^ spectra of precursor ions of the main lipid A species-related peak revealed that, as already reported for different Gram-negative bacteria [46], the lipid A of all examined strains is composed by a mixture of pyrophosphorylated (at position 1) and *bis*-phosphorylated species. This implies that LpxT is not the only *E. coli* protein able to perform lipid A pyrophosphorylation. This is in contrast with results previously reported by other authors showing the absence of the *tris*-phosphorylated lipid A species in Δ*lpxT* mutants [12,19,21,60]. This discrepancy might be due to differences in the experimental approaches and/or in the culture growth conditions. However, in support to our finding, pyrophosphorylation of the reducing glucosamine unit of the lipid A has been found also in *Bordetella bronchiseptica* [61] that actually lacks an LpxT orthologue, suggesting that other proteins can perform such activity.

## Figures and Tables

**Table 1:** Bacterial strains and plasmids.

**Strain**	**Genotype**	**Reference**
BW25113	*Escherichia coli* K12 derivative; F^-^ DE(*araD-araB*)*567 lacZ4787*(del)::*rrnB-3 LAM- rph-1* DE(*rhaD-rhaB*)*568 hsdR514*	[22]
KG-276	BW25113 *lpxT-sfGFP*	[17]
KG-279	BW25113 Δ*lpxT*	[17]
AMM05	BW25113 Δ*ldtD*	[23]
AMM06	BW25113 Δ*ldtE*	[23]
AMM07	BW25113 Δ*ldtD* Δ*ldtE*	[23]
**Plasmids**	**Relevant Characteristics ^a^**	**Origin/Reference**
pGM930	pBAD24-Δ1 [24] derivative; confers ampicillin resistance	[25]
pGZ119HE	cloning vector; confers chloramphenicol resistance	[26]
pLPXT	pGM930 derivative, carries the *lpxT* gene (2268826-2269744)	[17]
pLPXT^H190A^	pLPXTp derivative, carries the substitution of CAC codon at position 570 with GCC	This work
pLPXT-GFP	pGM930 derivative, carries the *lpxT*-GFP translational fusion	[17]
pUPPS	pGZ119HE derivative, carries the *uppS* gene (2542505-2543271)	This work

^a^*E. coli* coordinates refer to Genbank accession number U00096.3.

**Table 2:** Titer and optical density of overnight cultures carrying pLPXT.

Strain ^b^	+ARA ^a^			−ARA ^a^		
	OD_600_^c^	−amp ^d^	+amp ^d^	OD_600_^d^	−amp ^d^	+amp ^d^
BW25113/pLPXT	2.2 ± 0.2	130 ± 47	1.9 ± 0.8	3.0 ± 0.1	280 ± 14	260 ± 99
Δ*ldtD*/pLPXT	1.2 ± 0.2	230 ± 260	1.3 ± 0.2	3.0 ± 0.4	230 ± 90	290 ± 110
Δ*ldtE*/pLPXT	1.4 ± 0.4	150 ± 58	2.2 ± 2.5	3.1 ± 0.3	250 ± 26	210 ± 89
Δ*ldtDE*/pLPXT	1.8 ± 0.2	110 ± 35	2.8 ± 1.3	3.4 ± 0.0	220 ± 68	270 ± 50

^a^ A total of 5 mL of LD+100 µg/mL amp and with (+ARA) or without (−ARA) 0.01% arabinose were inoculated with single colonies and incubated 16 h at 37 °C, aerated, before reading the OD_600_. Aliquots were diluted and plated on LD10 with (+amp) or without (−amp) ampicillin. Values are the average ± standard deviation of the OD_600_ and titers (in cfu/mL; ×10^7^) of five (+ARA) or three (−ARA) independent cultures. ^b^ Δ*ldtD*/pLPXT, AMM05/pLPXT; Δ*ldtE*/pLPXT, AMM06/pLPXT; Δ*ldtDE*/pLPXT, AMM07/pLPXT. ^c^ Significance of the difference among reported OD_600_ was evaluated with ANOVA and Tukey’s post-hoc test. BW25113/pLPXT vs. Δ*ldtD*/pLPXT, *p* < 0.0001; BW25113/pLPXT vs. Δ*ldtE*/pLPXT, *p* < 0.01; Δ*ldtD*/pLPXT vs. Δ*ldtDE*/pLPXT, *p* < 0.05. Difference was not significant for all other comparisons. ^d^ Differences among the values in the column were not significant according to the ANOVA test.

## Supplementary Materials

The following are available online at https://www.mdpi.com/2076-2607/8/6/826/s1, Figure S1: Growth of cells carrying pGM930. Figure S2: Effect of glucose and *uppS* ectopic expression on the growth of *lpxT* overexpressing cells. Figure S3: Distribution of LPS in sucrose gradient fractions. Figure S4: MALDI-TOF MS analysis of isolated lipid A. Figure S5: Growth of cultures overexpressing *lpxT*^H190A^. Table S1: The main ion peaks in the MALDI-TOF MS spectrum reported in Figure S4 and the proposed interpretation of the substituting fatty acids on the lipid A backbone.
