# Supplementary material for: Overexpression of lpxT Gene in Escherichia coli Inhibits Cell Division and Causes Envelope Defects without Changing the Overall Phosphorylation Level of Lipid A
Source: Microorganisms. 2020 May 30;8(6):826. doi: 10.3390/microorganisms8060826 (PMC7356881; doi:10.3390/microorganisms8060826)
Supplement: Supplementary file 1 [file microorganisms-08-00826-s001.pdf]

Overexpression of *lpxT* Gene in *Escherichia coli* Inhibits Cell Division and Causes Envelope Defects Without Changing the Overall Phosphorylation Level of Lipid A

This file contains:

Figure S1. Growth of cells carrying pGM930

Figure S2. Effect of glucose and *uppS* ectopic expression on the growth of *lpxT* overexpressing cells

Figure S3. Distribution of LPS in sucrose gradient fractions.

Figure S4. MALDI-TOF MS analysis of isolated lipid A

Table S1. The main ion peaks in the MALDI-TOF MS spectrum reported in Figure S4 and the proposed interpretation of the substituting fatty acids on the lipid A backbone.

Figure S5. Growth of cultures overexpressing *lpxT*^{H190A}

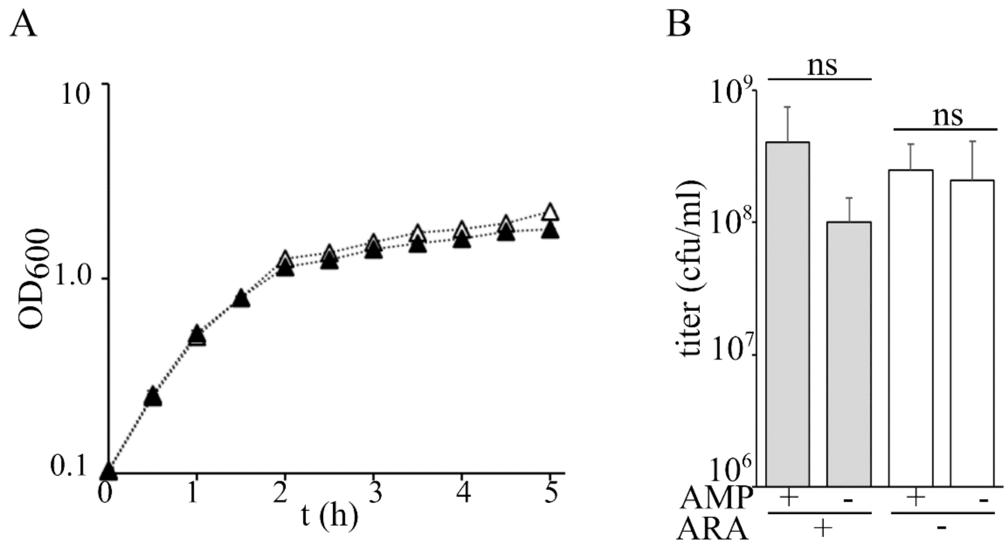

Figure S1. Growth of cells carrying pGM930. BW25113/pGM930 was inoculated in LD with 100 µg/mL ampicillin with (full symbols) or without (empty symbols) 0.01% arabinose and incubated at 37 °C monitoring the OD_{600}. (**A**). Each point represents the average of the OD_{600} of three independent cultures with relative standard deviation. Error bars are hidden by the symbols. (**B**). After 16 h incubation with (grey bars) or without (white bars) arabinose, cultures (n = 3) were serially diluted and plated on LD with (+AMP) or without (-AMP) ampicillin to measure viable counts. Columns represent average with standard deviation. Significance was estimated with t-test (ns, not significant).

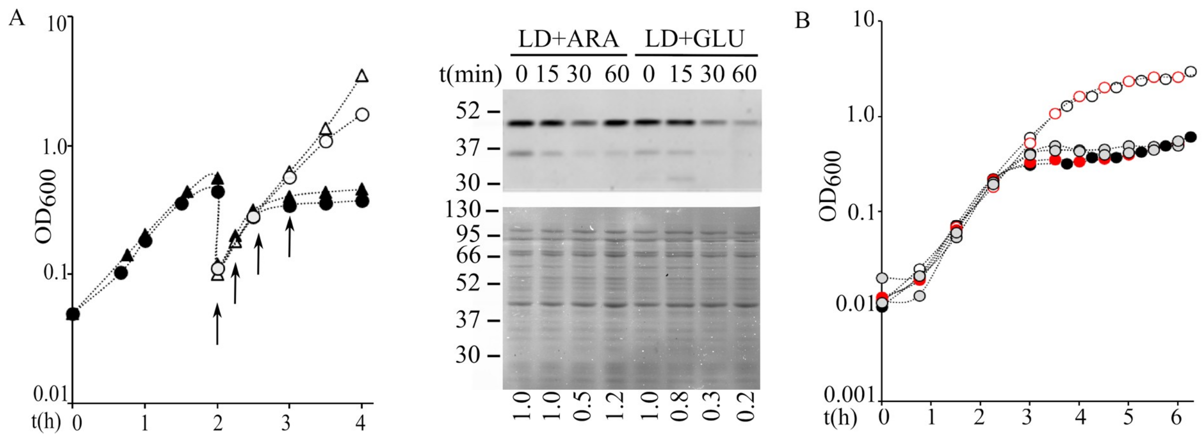

Figure S2. Effect of glucose and *uppS* ectopic expression on the growth of *lpxT* overexpressing cells. A. BW25113/pLPXT (circles) and BW25113/pLPXT-GFP (triangles) cultures were grown as described in LD with ampicillin and arabinose until the growth arrest and diluted to OD_{600} = 0.1 in LD with arabinose (black symbols) or glucose (empty symbols). All media contained 100 µg/mL ampicillin. (**A**), right panel. LpxT-sfGFP amount as estimated by in-gel fluorescence. Proteins extracted at the time points indicated by arrows in left panel were fractionated by 12% SDS-PAGE. Upper panel, in-gel fluorescence imaging; lower panel, gel stained with Coomassie. The numbers below the lanes refer to quantification with ImageLab (Biorad) of $LpxTsf_{GFP}$ signals normalized for the signal at *t* = 0 in LD+glucose. (**B**). Cultures of BW25113 carrying pLPXT and either pGZ119HE (black symbols) or pUPPS (red and grey symbols) were inoculated in LD 100 µg/mL ampicillin and 30 µg/mL chloramphenicol and with (filled symbols) or without (empty symbols) 0.01% arabinose and incubated at 37 °C reading the optical density at intervals. Representative results obtained in at least three replicate experiments are reported. Addition of 0.5 mM IPTG to the cultures at either t = 0 or after 3.5 h did not modify growth (grey symbols).

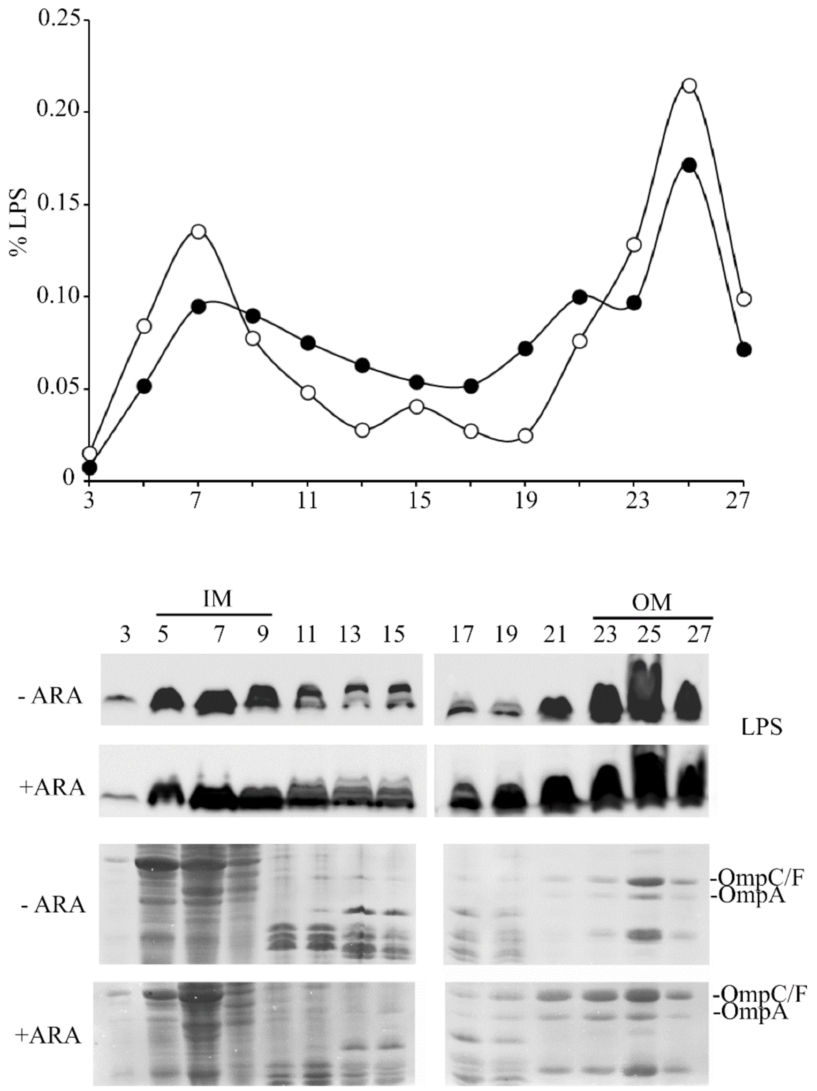

Figure S3. Distribution of LPS in sucrose gradient fractions. Cultures of BW25113/pLPXT were grown in LD with ampicillin up to OD_{600} = 0.2 and supplemented or not with 0.01% arabinose. Cells were collected 90 minutes after the induction (+ ARA) and at similar optical density for the uninduced (-ARA) culture (i.e. at ca. OD_{600} = 0.5–0.6). IMs and OMs were separated by isopycnic sucrose gradient centrifugation as described in Materials and Methods. Upper panel. LPS signals of induced (filled symbols) or uninduced (empty symbols) samples were quantified by densitometry with Image Lab (Biorad) and normalized for the total LPS signals. LPS distribution in fractions 1–19 *vs.* 21–27 was significantly different between induced and uninduced cells according to Pearson's chi-squared test (p <0.0001). Lower panels. LPS distribution across fractions was determined by Tricine-SDS-PAGE and immunoblotting using anti-LPS WN1 222-5 antibody. The profiles of the major OM porins (OmpC/F, OmpA) were determined by SDS-PAGE followed by Coomassie staining.

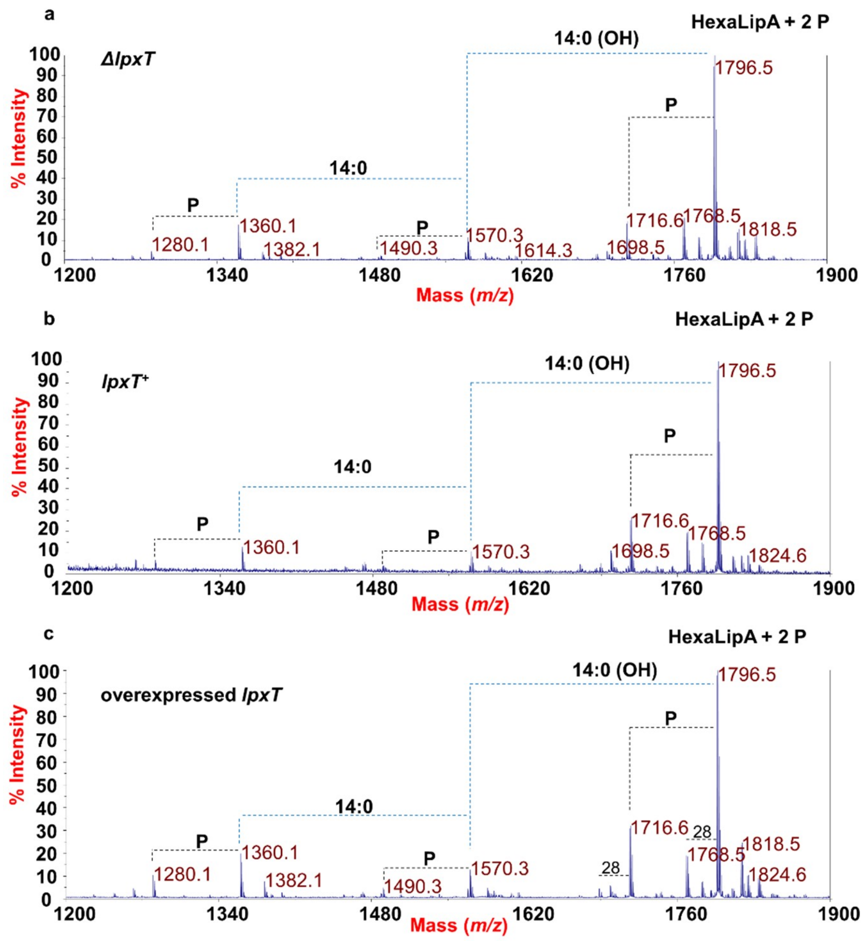

Figure S4. MALDI-TOF MS analysis of isolated lipid A. Negative-ion MALDI-TOF mass spectra of the isolated lipid A fractions from KG-279 (a, Δ*lpxT*), BW25113/pGM930 (b, *lpxT*+) and KG279/pLPXT (c, *lpxT* overexpressed) after mild acid hydrolysis of their isolated LPSs. Assignment of the lipid A species as HexaLipA (hexa-acylated lipid A species) and relative groups of ions lacking one phosphate, and/or one 14:0 (3-OH) and 14:0 acyl chains (namely penta- and tetra-acylated forms) is also indicated.

Table S1. The main ion peaks in the MALDI-TOF MS spectrum reported in Figure S4 and the proposed interpretation of the substituting fatty acids on the lipid A backbone.

Observed Ion Peaks (*m*/*z*)	Acyl Substitution	Proposed Fatty Acid/Phosphate Composition
1796.5	Hexa-acyl	$HexN^2P^2$ [14:0(3OH)]^4 (12:0) (14:0)
1716.6	Hexa-acyl	$HexN^2P$ [14:0(3OH)]^4 (12:0) (14:0)
1570.3	Penta-acyl	$HexN^2P^2$ [14:0(3OH)]^3 (12:0) (14:0)
1490.3	Penta-acyl	$HexN^2P$ [14:0(3OH)]^3 (12:0) (14:0)
1360.1	Tetra-acyl	$HexN^2P^2$ [14:0(3OH)]^3 (12:0)
1280.1	Tetra-acyl	$HexN^2P$ [14:0(3OH)]^3 (12:0)

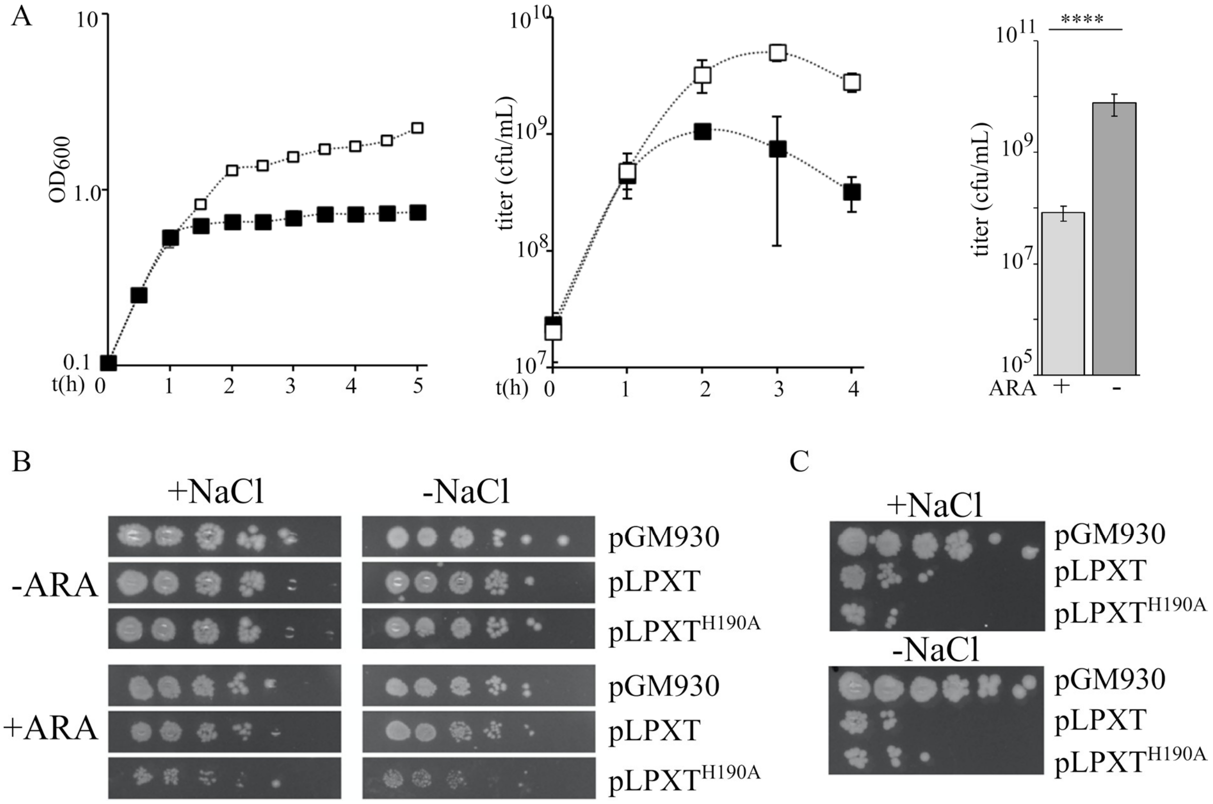

Figure S5. Growth of cultures overexpressing *lpxT*^*H190A*^. (**A**). Cultures of BW25113 carrying pLPXT^{H190A} were inoculated in LD with 100µg/mL ampicillin at OD_{600} = 0.05 and induced with 0.01% arabinose at OD_{600} = 0.2. Growth was followed by reading the OD_{600} with a spectrophotometer (left panel) or by plating samples on LD10 with ampicillin to measure viable counts (central panel). Empty symbols refer to uninduced cultures and black symbols to induced ones. Average values obtained in at least three replicate experiments are reported with standard deviation. In some cases, error bars are hidden by symbols. A, right panel. After 16h incubation with (+ARA) or without (-ARA) 0.01% arabinose, cultures were serially diluted and plated on LD with 100µg/mL ampicillin to measure viable counts. Significance was estimated with t-test *(****, p<0.0001).* (**B**). Cultures of BW25113 carrying the indicated plasmids were grown 16 h at 37 °C in LD with ampicillin, serially diluted (x10) and replicated on LD10 containing or not NaCl. The medium was supplemented with ampicillin and contained (+ ARA) or not (-ARA) 0.2% arabinose. The plates were incubated 20 h at 37 °C. C. Single colonies obtained in the diluted spots plated on LD10 containing (+NaCl) or not (-NaCl) salt were touched with a toothpick and resuspended in 0.1 mL of LD in a 96-well plate. The suspension was serially diluted (×10) and plated on LD10 with ampicillin. The experiment was repeated on 8 independent colonies for each strain with similar results.
